# Complex Behavior of Nano-Scale Tribo-Ceramic Films in Adaptive PVD Coatings under Extreme Tribological Conditions

**DOI:** 10.3390/e20120989

**Published:** 2018-12-19

**Authors:** German Fox-Rabinovich, Anatoly Kovalev, Iosif Gershman, Dmitry Wainstein, Myriam H. Aguirre, Danielle Covelli, Jose Paiva, Kenji Yamamoto, Stephen Veldhuis

**Affiliations:** 1 Department of Mechanical Engineering, McMaster University, 1280 Main St. W., Hamilton, ON L8S 4L7, Canada; 2 SPRG Surface Phenomena Researches Group, Physical Metallurgy Institute CNIICHERMET, 2nd Baumanskaya str., 9/23, off. 475, Moscow 105005, Russia; 3 Moscow State Technological University “Stankin” (MSTU “STANKIN”), Joint Stock Company Railway Research Institute, Moscow 127994, Russia; 4 LMA—Laboratory of Advanced Microscopy, at INA—Institute of Nanoscience of Aragón, University of Zaragoza, Edificio I + D. Campus Rio Ebro.C/Mariano Esquillor, s/n 50018 Zaragoza, Spain; 5 Saskatchewan Structural Sciences Centre, University of Saskatchewan, Thorvaldson Building Office 186, 110 Science Place, Saskatoon, SK S7N 5C9, Canada; 6 Kobe Steel Ltd., Kobe 651-2271, Japan

**Keywords:** tribo-ceramic films, self-organized systems, H13 ultra-speed milling machining

## Abstract

Experimental investigations of nano-scale spatio-temporal effects that occur on the friction surface under extreme tribological stimuli, in combination with thermodynamic modeling of the self-organization process, are presented in this paper. The study was performed on adaptive PVD (physical vapor deposited) coatings represented by the TiAlCrSiYN/TiAlCrN nano-multilayer PVD coating. A detailed analysis of the worn surface was conducted using scanning electron microscopy and energy dispersive spectroscopy (SEM/EDS), transmission electron microscopy (TEM), X-ray photoelectron spectroscopy (XPS), and Auger electron spectroscopy (AES) methods. It was demonstrated that the coating studied exhibits a very fast adaptive response to the extreme external stimuli through the formation of an increased amount of protective surface tribo-films at the very beginning of the running-in stage of wear. Analysis performed on the friction surface indicates that all of the tribo-film formation processes occur in the nanoscopic scale. The tribo-films form as thermal barrier tribo-ceramics with a complex composition and very low thermal conductivity under high operating temperatures, thus demonstrating reduced friction which results in low cutting forces and wear values. This process presents an opportunity for the surface layer to attain a strong non-equilibrium state. This leads to the stabilization of the exchanging interactions between the tool and environment at a low wear level. This effect is the consequence of the synergistic behavior of complex matter represented by the dynamically formed nano-scale tribo-film layer.

## 1. Introduction

The running-in period is the actual stage of wear when self-organization begins and progresses towards the post-running-in (stable stage) when wear rate stabilizes [1]. In the current research, extreme tribological conditions were achieved under ultra-speed ‘dry’ (no coolant) machining of hardened H13 tool steel (HRC 52–55). This process is considered to be high-performance machining, which can provide a high rate of material removal combined with good surface quality and dimensional accuracy of the workpiece material [2]. High-performance machining represents a unique case of an extreme external environment [3,4,5], due to a combination of high temperatures, around 1000–1300 °C, and pressures of 2–5 GPa [6,7] during high-speed cutting. As such, these tribological conditions are in a strong non-equilibrium state [8] and, thus, present an ideal case study of the self-organization process during friction [1], particularly with surface-engineered nano-material experiments [9,10,11]. 

The cutting tool actively adapts to the cutting conditions [12]. Self-organization during friction develops through tribo-film formation on the surface of the operating tool. In general, two kinds of tribo-films could be observed: (1) near-amorphous ductile and lubricious and (2) crystalline tribo-ceramics with thermal barrier properties, as well as increased hardness and strength [13,14,15]. Tribo-films of the first type are supersaturated solid solutions formed by reaction with the elements from the environment (mostly oxygen) [16,17]. They are distinguished by a high level of disordering. With the presence of these tribo-films, the material may become super-plastic (due to a nano-scale grain or amorphous microstructure) [16,17]. The amorphous microstructure of the tribo-films might also lower the thermal conductivity of the surface [16]. Tribo-films of the second type are usually non-stoichiometric compounds. Tribo-films of this type exhibit high thermodynamic stability, good thermal barrier properties, and high hardness. The entire tribo-film layer has a complex chemical composition during cutting and as was shown previously, an amorphous/crystalline nano-scale structure [18,19]. In this way, the tribo-film layer, to some degree, represents what is known as complex matter, which is a matter that has informed, self-organized, evolutive properties [20,21]. The word ‘informed’ is used in a sense to describe matter in which composition, microstructure, and transformation develops into increasingly sophisticated architectures and behaviors [20,22]. This paper features several such instances of complex matter behavior present in the dynamically-formed tribo-film layer.

This paper demonstrates an example of adaptive coating [23] represented by a nano-multilayer (TiAlCrSiYN/TiAlCrN PVD) coating, which is capable of sustaining severe operating conditions [24]. Upon interaction with the environment, all of the indicative features of adaptive coatings are manifested in the studied tribo-system, such as openness, non-equilibrium state, complexity, hierarchical transformations, and emergent performance [18,23,24,25]. The key part of this investigation is concerned with the complex interaction between a number of non-linear processes on the friction surface at the nano-scale, under outlined extreme tribological conditions.

## 2. Thermodynamic Modeling of the Tribo-Films Formation

Self-organization in tribo-systems leads to a significant decrease in wear rate. It has been shown in [1] for various tribo-systems, including the coated cutting tool. The earlier self-organization occurs in the tribo-system, the lower its integral wear will be. According to [26], the possibility of self-organization is determined by the sign of excessive entropy production. In this regard, we consider below the production and excessive entropy production, especially at the initial stage of running-in. 

As it was outlined above a tribo-system of one frictional body is considered, in particular a cutting tool. The volume of the system, as a rule, is limited by (1) area of interaction (contact area); (2) thickness of the secondary structures or tribo-films, which are new structures formed in situ on the friction surface.

The entropy production is:

(1) $$\frac{dS_{i}}{dt}=\sum_{i}X_{i}J_{i},$$

where: $\frac{dS_{i}}{dt}$ is the entropy production, $X_{i}$ are the thermodynamic forces, and $J_{i}$ are the thermodynamic flows.

If only the processes of friction and thermal conductivity are considered:

(2) $$J_{i}=-\lambda BgradT$$

(3) $$X_{i}=-\frac{l\left(gradT\right)}{T^{2}},$$

where λ is the coefficient of thermal conductivity of the surface layers of the cutting tool, where the interaction is concentrated; B is the area of contact; T is the average temperature of these layers; and l is the thickness of these layers.

Assuming that the entire heat flux in the process of friction goes into the cutting tool, then at the same time:

(4) $$J_{i}=kNv,$$

where k is the coefficient of friction; N is the average stress at the friction surface; and v is the cutting speed.

It follows from Equations (2) and (4):

$$gradT=\frac{-kNv}{\lambda B}$$

Consequently, if only the friction process is taken into account, the entropy production on the surface layer of the cutting tool will look like this:

(5) $$\frac{dS_{i}}{dt}=\frac{l{\left(kNv\right)}^{2}}{\lambda T^{2}B}$$

In Equation (6):

(6) $$J_{i}=kNv;X_{i}=\frac{l\left(kNv\right)}{\lambda T^{2}B}$$

According to [26], the necessary condition for the thermodynamic stability of a given system is:

(7) $$\frac{1}{2}\frac{\partial }{\partial t}\left(\delta^{2}S\right)=\underset{i}{\sum }\delta X_{i}\delta J_{i}\geq 0$$

where δ are the fluctuation and S is the entropy of the volume of the system.

The sum on the right is called the excessive entropy production. The quantities $X_{i}$ and $J_{i}$ are the deviations of the corresponding flows and forces in the stationary state. If, starting with the beginning of the perturbation, the inequality holds such that:

(8) $$\frac{\partial }{\partial t}\delta^{2}S\geq 0$$

then this state is stable. However, under certain processes or their various interactions, it is possible to obtain a negative contribution to the excess production of entropy, which grows with increasing perturbation. In such case, this state can become unstable because the positive excess production of entropy is a necessary, but not sufficient condition of stability. Only after passing through the instability, the process of self-organization can begin. Thus, the necessary condition for the loss of stability and hence the beginning of self-organization is:

(9) $$\frac{\partial }{\partial t}\delta^{2}S<0$$

In this system, the excess production of entropy is as follows:

(10) $$\frac{\partial }{\partial t}\delta^{2}S=\delta kNv\delta \frac{l\left(kNv\right)}{\lambda T^{2}B}$$

**Figure 1:**
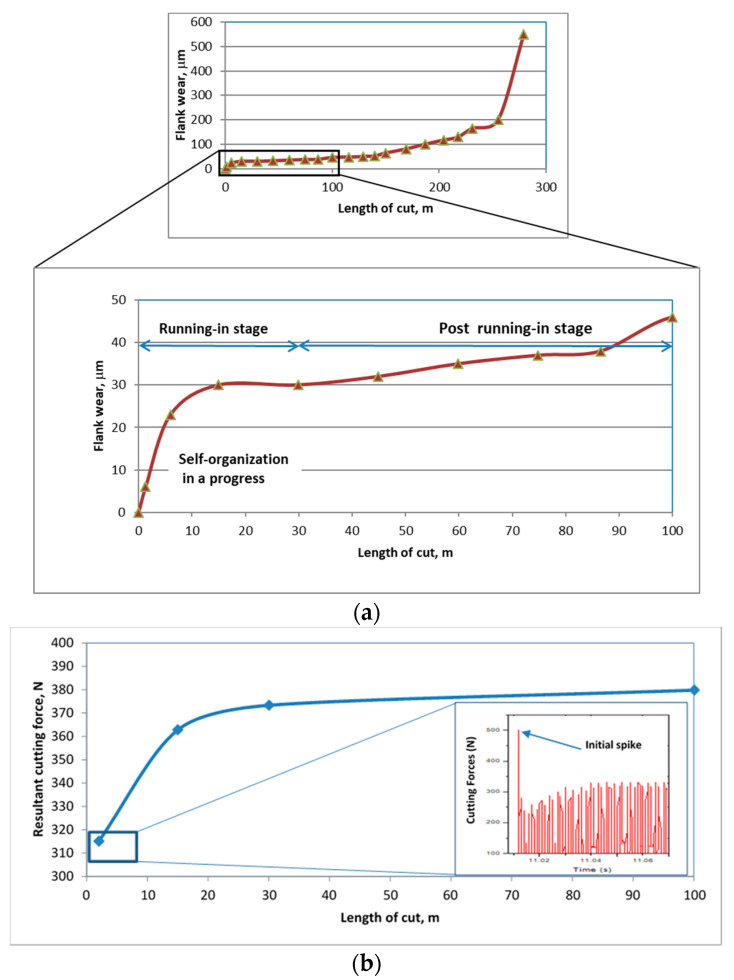
Flank wear vs. length of cut data for C-2SB ball nose end mills: (**a**) wear curve with an indication of specific stages of wear: running-in, post-running-in, stable stage of wear; and (**b**) cutting forces data.

At the beginning of the cutting process (Figure 1a), the cutting speed and the stress value remain constant. The tribological forces, in this case, represented by the cutting forces [27], vary (Figure 1b) depending on the friction conditions in the cutting zone (cutting force spike—Figure 1b).

It will be assumed that the thickness of the secondary structures (*l*), the coefficient of thermal conductivity (*λ*), the contacting area (*B*) and the average temperature (*T*) all depend on the tribological forces, which can be evaluated by the cutting force (*N*) and the cutting speed (*v*) will be assumed to be constant. Then Equation (10) can be rewritten in the form:

(11) $$\frac{\partial }{\partial t}\left(\delta^{2}S\right)={\left(Nv\right)}^{2}\left(\frac{l}{\lambda BT^{2}}+\frac{k}{\lambda BT^{2}}\frac{\partial l}{\partial k}-\frac{lk}{\lambda^{2}BT^{2}}\frac{\partial \lambda }{\partial k}-\frac{lk}{\lambda T^{2}B^{2}}\frac{\partial B}{\partial k}-\frac{2kl}{\lambda BT^{3}}\frac{\partial T}{\partial k}\right){\left(\delta k\right)}^{2}$$

The first factor on the right-hand side of Equation (11) is positive (quadratic form). Equation (11) can become negative if the second factor on the right-hand side of Equation (11) is negative. The first term of the second factor on the right side of Equation (11) is positive. It is most likely that the second factor on the right-hand side of Equation (11) can become negative if the remaining terms become negative. This can happen under the following conditions:

(12) $$\frac{\partial l}{\partial k}<0;\frac{\partial \lambda }{\partial k}>0;\frac{\partial B}{\partial k}>0;\frac{\partial T}{\partial k}>0$$

The first inequality in Equation (12) $\frac{\partial l}{\partial k}<0$ describes the decrease in the thickness of the surface structures formed before friction. In fact, this inequality represents the “annihilation” of surface structures during friction that were formed prior to it. Such effects are known [28]. This means that the tribo-system primarily “destroys” the initial surface structures prior to the formation of secondary structures (tribo-films). In this case, the observance of the first inequality in Equation (12) becomes quite natural, especially if adhesive interaction occurs.

The second inequality in Equation (12) $\frac{\partial \lambda }{ðk}>0$ describes an increase in the thermal conductivity of the surface layers at the beginning of friction. This, as in the case of the first inequality, can also be associated with the “destruction” of surface structures during friction that formed prior to it. In addition, this inequality can hold true during the adhesive interaction, which is typical for the initial period of cutting.

The third inequality in Equation (12) $\frac{\partial B}{\partial k}>0$ can be a consequence of the adhesive interaction at the initial stage of the cutting. During the adhesive interaction, the real contact area grows, since contact occupies virtually the entire area between the asperities.

The fourth inequality is completely natural. The temperature grows with increasing coefficient of friction.

All four inequalities in Equation (12) are true if it is assumed that the frictional forces are increasing. Figure 1b shows the increase in the cutting forces over the entire length of cut. The most intensive growth of frictional forces is observed at the initial stage. The initial spike on the cutting forces (Figure 1b), is the result of the frictional condition on the cutting zone [27]. Thus, considering the parametric dependence of the frictional (cutting) force, obtained during on the length of cut or on the cutting time (*t*) then the inequalities in Equation (12) will look like this:

(13) $$\frac{\partial l}{\partial k}\frac{\partial k}{\partial t}<0;\frac{\partial \lambda }{\partial k}\frac{\partial k}{\partial t}>0;\frac{\partial B}{\partial k}\frac{\partial k}{\partial t}>0;\frac{\partial T}{\partial k}\frac{\partial k}{\partial t}>0$$

According to Figure 1b, the derivative $\frac{\partial k}{\partial t}$ for the cutting forces will be maximal at the beginning of friction, when the chip formation starts. Consequently, the greatest probability of the system to undergo the self-organization process is noted at the beginning of friction.

Now it will be shown that the formation of secondary structures during the initial stage of friction occurs with the greatest speed. The main mechanisms for the formation of secondary structures are mass transfer and chemical reactions. More precisely, the main mechanisms of mass transfer near the friction surface are plastic deformation and diffusion.

Starting from general considerations, it can be said that part of the production of entropy due to plastic deformation is the product of the flow caused by the thermodynamic forces. The volume of matter subject to plastic deformation will be considered to be the flow. Thus, the thermodynamic force will be mechanical stress exceeding the yield strength:

(14) $$\frac{d_{i}S_{d}}{dt}=kN\frac{dV}{dt},$$

where the index *S_d_* denotes plastic deformation and *V* the volume of a substance subject to plastic deformation.

Suppose that:

(15) V=Bh,

where *h* is the thickness of the deformed material, which grows from the beginning of friction.

Figure 1 presents data on (a) flank wear and (b) cutting forces (frictional conditions) vs. the length of cut. It was noted above that among observable cutting characteristics, the changes of the frictional forces are the most clear (Figure 1b). In this case *h* = *h*(*k*). With this in mind Equation (14) can be rewritten as follows (assuming that the contact area “*B*” has not changed):

(16) $$\frac{d_{i}S_{d}}{dt}=kNB\frac{dh}{dk}\frac{dk}{dt}$$

It follows from Figure 1b that the most intensive growth of the cutting forces is observed at the beginning of friction, hence the greatest intensity of plastic deformation will also be at the beginning of friction. The mass transfer rate due to diffusion is proportional to the difference of chemical potentials on both the friction surface and between the near-surface layers. In a limited volume, diffusion develops as a competition of various elements. Similar speculation for plastic deformation leads to the similar result. The most intense diffusion occurs during the beginning of friction. Therefore, the formation of secondary structures (tribo-films) occurs most intensively at that point as well. As a result of the adaptive response of the coated cutting tool/workpiece tribo-system, a large amount of wear protective and thermal barrier tribo-ceramics begin to generate on the friction surface, leading to the subsequent cutting force decrease. 

## 3. Experimental

An advanced TiAlCrSiYN/TiAlCrN multilayer coating was studied. The nano-multilayered Ti_0.2_Al_0.55_Cr_0.2_Si_0.03_Y_0.02_N/Ti_0.25_Al_0.65_Cr_0.1_N coating was deposited using Ti_0.2_Al_0.55_Cr_0.2_Si_0.03_Y_0.02_ and Ti_0.25_Al_0.65_Cr_0.1_ targets correspondingly fabricated by a powder metallurgical process on cemented carbide ball nose end mills WC-Co substrate in a R and D-type hybrid PVD coater (Kobe Steel Ltd. Hyogo, Japan) by a plasma-enhanced arc source. Samples were heated up to about 500 °C and cleaned through Ar ion etching process. Ar-N_2_ mixture gas was fed to the chamber at a pressure of 2.7 Pa with a N_2_ partial pressure of 1.3 Pa. The arc source was operated at 100 A for a 100 mm diameter ×16 mm thick target. The other deposition parameters were: bias voltage 100 V; substrate rotation 5 rpm. The thickness of the coating studied was around 3 µm for the film characterization and cutting tool test. The coating has a nano-crystalline/multilayered microstructure with alternating nano-layers period of 20–30 nm [24,25] and hardness of 30 GPa (measured using the nano-indentation method at room temperature and 28 GPa at elevated temperatures of 500 °C [3]. The measurements were performed using MicroMaterials NanoTest System (Wrexhan, UK). The experiments were designed as load controlled experiments. Trial tests were performed to determine a maximum load to give measurements at around 1/10 coating thickness. Based on these, a load of 40 mN was selected as this produced indentation contact depth of ~300 nm. The other coating mechanical properties such as elastic modulus and impact fatigue fracture resistance were also studied in detail [24]. Oxidation resistance of the studied coating vs. temperature in air has been investigated by using Thermogravimetric–Differential Thermal Analysis (TG-DTA) Model EV02 Rigaku (Akishima-shi, Tokyo, Japan) [25]. Only a very low weight gain of the multi-layered coating under the operating temperatures during the cutting test was observed (TG data). DTA data determines the thermal behavior of the coating and indicates on formation of oxide films on the surface that results in improvement of the thermal performance of the friction surface. DTA drastically reduces with temperature for the multi-layered coating studied [25]. Improved thermal behavior promotes beneficial heat dissipation via chip removal and prevents overheating of the coated tool surface.

To investigate the coatings on the cemented carbide WC/Co substrates, cross-sectional TEM observation was employed in combination with FIB (focused ion beam). Transmission electron microscopy and selected area electron diffraction (SAED) were performed in a JEOL FS2200 (Akishima-shi, Tokyo, Japan) microscope at an acceleration voltage of 200 kV.

The microstructural and phase transformation at the cutting tool/workpiece interface as well as the chemical composition of the tribo-films formed was studied with X-ray photoelectron spectroscopy (XPS) on a Physical Electronics (PHI) Quantera II spectrometer (Chanhassen, MN, USA) equipped with a hemispherical energy analyzer and an Al anode source for X-ray generation and a quartz crystal monochromator for focusing the generated X-rays. A monochromatic Al K-α X-ray (wavelength of 0.834 nm) source was operated at 50 W, 15 kV. The system base pressure was no higher than 1.0 × 10^−9^ Torr with an operating pressure that did not exceed 2.0 × 10^−8^ Torr. Before any spectra were collected from the samples, the samples were sputter-cleaned for 4 min using a 4 kV Ar+ beam. A 200 micron beam was used for all data collected on the samples. Pass energy of 280 eV was used to obtain all survey spectra while pass energy of 69 eV was used to collect all high-resolution data. All spectra were obtained at a 45˚ takeoff angle and utilized a dual beam charge compensation system to ensure neutralization of all samples. The instrument was calibrated using a freshly cleaned Ag reference foil, where the Ag 3d5/2 peak was set to 368 eV. All data analysis was performed using PHI Multipak version 9.4.0.7 software. 

Cutting tests were performed during dry ball nose end milling (Mitsubishi carbide ball nose end mills, D = 10 mm) of the hardened H13 tool steel with hardness HRC 52–55. The cutting experiments were carried out on a three-axis vertical milling center (Matsuura FX-5, Fukui, Japan). The cutting parameters were as follows: speed 500–700 m/min; feed: 0.06 mm/tooth; axial depth: 5.0 mm; and radial depth: 0.6 mm. The coated tool flank wear was measured using an optical microscope (Mitutoyo model TM, Kawasaki, Japan). A tool dynamometer (9255B, Kistler, Winterthur, Switzerland) was used to measure the cutting forces. At least three cutting tests were performed under corresponding operations. The scatter of the tool lifetime measurements was approximately 10%. 

## 4. Results and Discussion

### 4.1. The Accelerated Adaptive Response of the Coating during the Initial Stage of Running-In Processes

**Figure 2:**
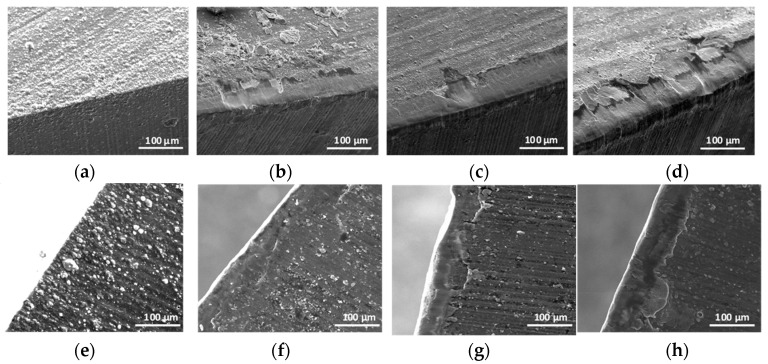
SEM images of the worn surfaces of C-2SB ball nose end mills: rake surface in the initial state (**a**) after 2 (**b**); 15 (**c**) and 30 m (**d**) correspondingly. The worn flank surface is presented in (**e**) initial state; (**f**) after 2 m; (**g**) 15 m; and (**h**) 30 m length of cut, correspondingly.

**Figure 3:**
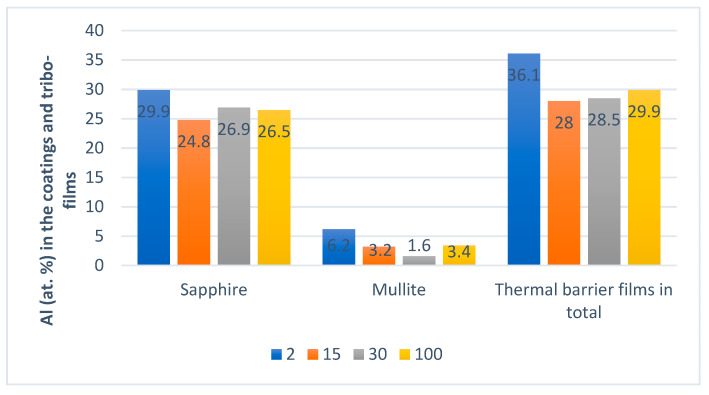
XPS data on the tribo-films formation vs. length of cut. Amount of tribo-films vs. the length of cut: 2; 15; 30; and 100 m.

Flank wear vs. length of cut data for C-2SB ball nose end mills is presented in Figure 1a. It shows that the wear value during the running-in stage is not high, but it is growing fast (Figure 1a). SEM images of the worn surfaces are presented in Figure 2. Only slight changes in wear patterns were observed on the flank and rake surfaces of the tool. XPS data (Figure 3) demonstrate an increased amount of protective tribo-ceramic films. They form as a consequence of an adaptive response of the tribo-system to external stimuli. At the initial stage (before cutting), the amount of oxide tribo-films does not exceed 1%–2%. Note that the highest total amount of tribo-ceramics forms only after 1 min of cutting (length of cut of 2 m, which is around 1%–2% of the entire cutting time before the end of tool lifetime) leading to a minimum value of cutting forces (Figure 1b). This is consistent with the thermodynamic modeling results where the greatest probability of self-organization in this tribo-system is achieved at the initial stage of cutting.

**Figure 4:**
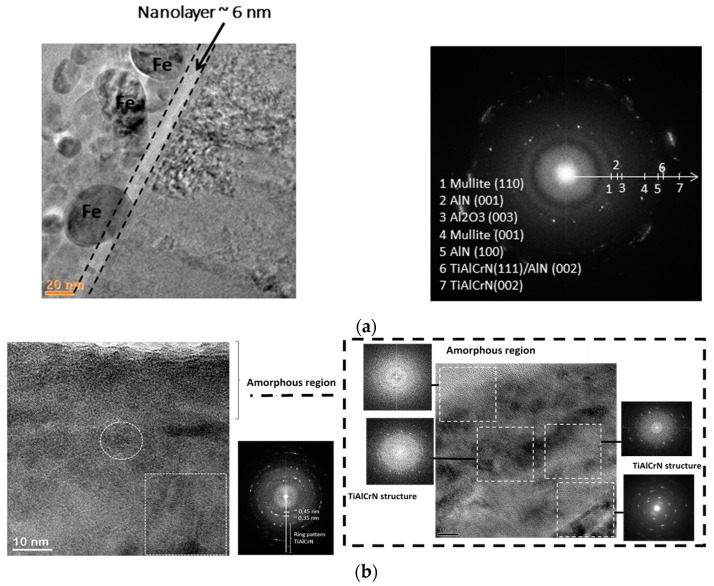
HRTEM image of the FIB cross-section of the worn surface, 15 m length of cut: (**a**) nano-layer of tribo-films on the surface of TiAlCrSiYN/TiAlCrN coating (6 nm thick); and (**b**) amorphous surface coating layer.

Direct TEM observation shows that the layer of dynamically replenishing tribo-films is in the nano-scale range (thickness of around 6 nm) (Figure 4a), which corresponds to the previous obtained data [29]. They form the layer, which has an amorphous/crystalline microstructure. The present data illustrate how quickly a properly designed surface-engineered layer (such as a nano-multilayer TiAlCrSiYN/TiAlCrN coating) can efficiently adapt to extreme external stimuli. Due to a highly non-equilibrium state [30] and complex nano-crystalline/laminated structure [24], the TiAlCrSiYN/TiAlCrN coating can boost spontaneous predominant mass transfer of necessary elements, primarily Al and Si to the friction surface. The relationship of these spontaneous processes to the non-spontaneous processes of tribo-oxidation, followed with the formation of dissipative structures, eventually leads to a strong decrease in entropy production and corresponding wear rate as a result of self-organization within the very initial stage of wear (Figure 1). 

Once the process of friction has been stabilized, then the entropy production remains very small in the post running-in stage, (after a length of cut above ~30 m), due to permanent tribo-film replenishment [3]. This leads to sustainable surface protection through a constant amount of tribo-films forming on the friction surface (Figure 3).

Figure 4 presents the microstructure of a worn tool surface. The tribo-oxide layer is around 6 nm thick. According to the SAED pattern (Figure 4a on the right), it has a complex phase composition and contains a mixture of many non-equilibrium oxides. 

Such oxides are formed by non-spontaneous processes accompanied by negative entropy production. The development of such processes is a key feature of self-organization, which, as shown in Section 2, is most likely to commence at the beginning of the cutting process.

These tribo-ceramic films protect the underlying layers of the nitride coating from oxidation but does not prevent plastic deformation of the layer. As can be seen in Figure 4b (directly below the tribo-film), the layer of the nitride coating is amorphized up to a thickness of about 20–25 nm. Electron diffraction patterns in Figure 4b reveal that the micro-structure of the coating becomes amorphous/nano-crystalline at this level. The layers of the nitride coating retain their crystalline microstructure at a greater thickness (electron diffraction patterns in Figure 4b). Within the wear zone, phase and structural transformations are extremely complex and multi-stage. During this initial period of cutting, the dynamic process of tribo-oxidation develops. In this way, the surface of the nitride layer is protected against overheating and damaging oxidative wear [25] is avoided. Due to the heat-protective properties of the thinnest tribo-ceramic films with low thermal conductivity at elevated temperatures of cutting [31,32,33], no recrystallization of the amorphized nitride coating sublayer is observed. 

The complex composition and amorphous/nano-crystalline microstructure of the tribo-ceramic nano-layer may significantly impact its thermal barrier/energy dissipative characteristics [13]. Both crystalline thermal barrier ceramics (such as sapphire or mullite) [31] and amorphous-like phases [32] strongly contribute to the reduction of the thermal conductivity of the layer. 

**Figure 5:**
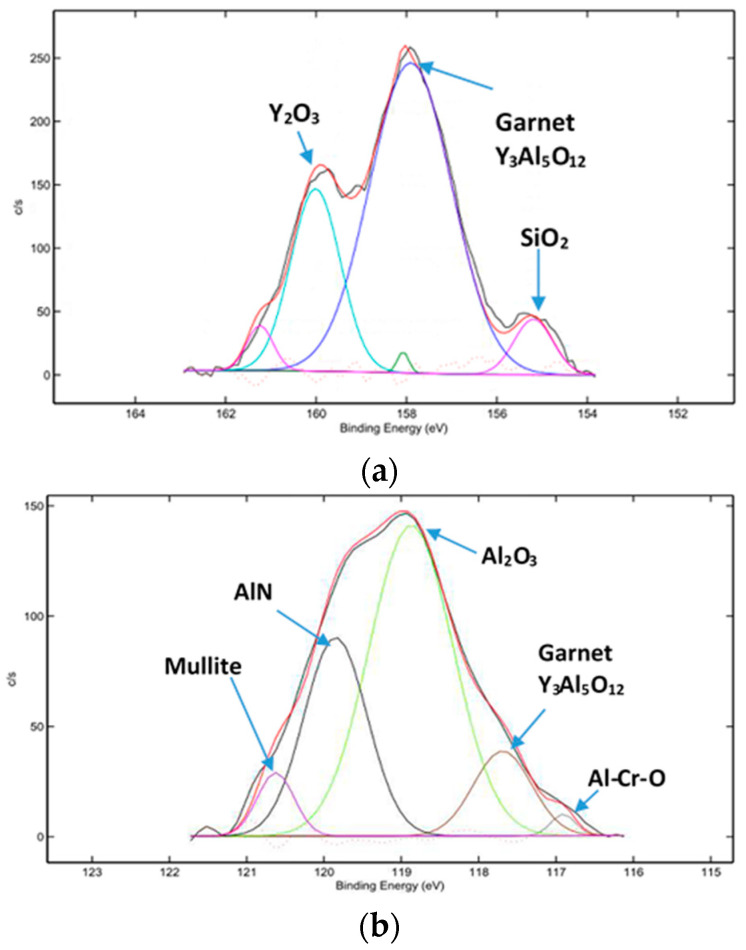
HR XPS spectra of complex tribo-oxides formed the beginning of running-in stage: (**a**) the Y 3d spectrum; and (**b**) the Al 2s spectrum.

Moreover, previously published data indicate the formation of elementary Ti, Al, Cr, Si, and Y tribo-oxides on the friction surface of the TiAlCrSiYN/TiAlCrN coating [24,30]. Most significantly, all of these ‘primary’ tribo-oxides interact with each other during wear, further forming complex oxide films such as Al_6_Si_2_O_13_ mullite [24]; Al-Cr-O tribo-phase [30,31] and even garnet [32] tribo-phases (see Figure 5). These complex oxides have reduced thermal conductivity at high operating temperatures compared to the major tribo-phase (sapphire) [29,31,32,33]. It is very important for the alumina to have a negative temperature dependence on the thermal conductivity coefficient, thus protecting the initial coating from heat damage [31]. Formation of such complex tribo-oxides significantly improves surface protection. The tribo-oxides are the products of a non-spontaneous, non-linear process, which leads to the decrease of entropy production in the system during the running-in stage of wear [34].

The phenomena involved in this process determine the performance of the tribo-films. Friction at the tool/chip interface repeatedly generates a very intensive heat flux. This heat flux should be mostly transferred to the environment due to the very low heat conductivity of the nano-scale layer of the tribo-oxides [35]. Most of the thermal protection is due to the mean free path of phonons within the nano-layer of the tribo-oxides reaching or exceeding the thickness of the tribo-oxide films [36]. This blocks thermal transport through the tribo-film layer. The scattering of phonon oscillations throughout numerous defects and interfaces is another reason for the loss of heat transfer through tribo- films [33]. Another contributing factor is the high density of lattice defects generated by high pressure on the friction surface under extreme conditions [29]. All of these factors work together to result in enormously high thermal barrier properties developing at the nano-scale, right within the tribo-film layer [3]. Therefore, for the most part, heat cannot transfer into the body of the coating layer. Instead, it dissipates via chip removal and radiation into the surrounding environment. 

This feature of the tribo-film layer strongly promotes heat dissipation and results in a dramatic temperature gradient within the nano-scale layer of tribo-films [37,38]. The proposed mechanism of heat dissipation at the nano-scale prevents damage of the tribo-film/coating interface with unprecedented efficiency and consistency [3]. As a result, a very strong temperature gradient was achieved under extreme operational conditions (speed of 500 m/min), significantly reducing the temperature beneath the thermal barrier/lubricating nano-layer of the tribo-films as it was clearly shown by TEM studies of the worn coating layer [3]. The temperature below the nano-scale layer of the tribo-films drops by a few hundred degrees C (estimated at around 400 °C) [37]. This results in excellent protection of the friction surfaces and overall performance control of the entire surface engineered system [39,40]. The phenomenon observed can be contingently called a ’heat flow reflection effect’. This effect involves many simultaneously developing processes outlined above and is strongly associated with synergistic, emergent-like behavior of complex matter [20].

It was remarked in the thermodynamic model that the system has the greatest probability of self-organization at the very initial stage of wear. Experimental analysis of the coated cutting tool surface confirmed that the self-organization processes developed at the initial stage of wear. Self-organization is a probabilistic process. If it does not transpire during the initial stage, the wear rate would be as high as after 10 m of cutting. The wear rate is characterized by the tangent of slope of the curve in Figure 1a. Intensive self-organization (with the formation of dissipative structures, i.e., tribo-films) generally ends at a cutting length of 10–15 m, when wear rate sharply drops. If intensive self-organization does not transpire at the very beginning of cutting, but later the total wear of the tool would significantly increase, just as a sharp drop in the wear rate occurs only after a longer cutting length. 

### 4.2. Spatio-Temporal Behavior of Tribo-Films during Various Stages of Wear

**Figure 6:**
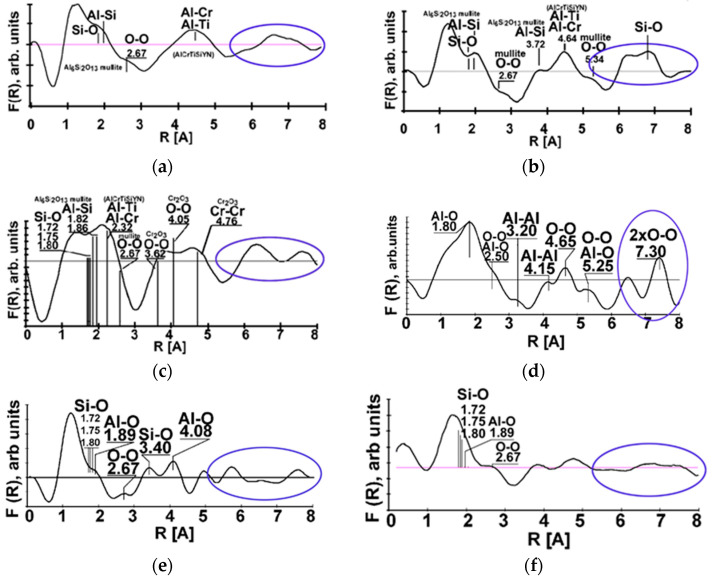
The evolution of the atomic coordination in the secondary oxide films after different cutting length: (**a**) 2 m length of cut; (**b**) 15 m length of cut. The blue oval indicates the long-range order region (EELFS Fourier transforms); (**c**) 30m cutting length, mullite-like tribo-oxides; (**d**) 60 m cutting length, sapphire-like tribo-oxides; (**e**) mullite-like and amorphous after length of cut of 100 m; and (**f**) tribo-oxides after length of cut of 100 m.

One more feature of tribo-film behavior may be introduced. As was outlined above, the tribo-films observed in this study under extreme cutting conditions are a mixture of two types of tribo-films (amorphous and crystalline) known from general tribology [1]. In this case, they have an amorphous-crystalline structure and represent a mixture of the two kinds of tribo-films presented in Figure 6 as proved by Fourier transforms. It was reported in [13,22] that separate islands of non-equilibrium nitrides and oxides are formed during the initial stage of wear. At the beginning of the running-in stage of wear (cutting length of 2 m), the oxide thickness was about 1.8 nm and had an amorphous structure according to electron energy losses fine structure (EELFS) (Figure 6a) [7]. Only the nearest coordination spheres with radii up to 0.4 nm are forming the peaks. One can see in Figure 6b that a mixture of Al and Si oxides with a mullite-like structure is formed after a length of cut of 15 m. The intensity of the F(R) characteristic is slightly increased. These details are indicated with the blue ovals in Figure 6. After a length of cut of 15 m, nano-crystalline/amorphous oxides form on the friction surface. After a length of cut of 30 m, tribo-oxidation provides an increased variety of oxides on the worn surface (Figure 6c,d). The sapphire-like Al_2_O_3_ and Cr_2_O_3_ tribo-oxides with a large amount of structure defects were also observed. The thickness of the tribo-film grows from 1.8 up to 6 nm [7]. The sapphire-like tribo-films were observed after 60 m of cutting (Figure 6d). These tribo-films are characterized by a stronger long-range order in 0.4–0.8 nm region of the nearest atomic surrounding as compared to the mullite and chromium oxides after 30 m of cutting (Figure 6c).

At the end of the running-in stage and during the stable stage of wear, the oxide tribo-films cover the “tool-chip” contact area. Wear velocity of the oxide films is very low due to their potent mechanical and protective properties and high temperatures that develop during high-speed dry cutting. This leads to recrystallization processes within the layer of the oxides [41].

However, once the process develops in time (within the steady stage of wear), the atomic structure of the tool surface begins to change again (see Figure 6e,f), after 100 m of cutting): EELFS at a small (less than 1 µm) beam size, reveals areas with an amorphous structure similar to the one present in Figure 6a. The long-range order of the tribo-films disappears, and their structure again becomes amorphous.

As mentioned in [37], these amorphous zones form due to the accumulation of damage in the upper layers of the nitride coating (Figure 4a) and localized detachment of the “old” oxides. Fresh amorphous oxides form on the open surface of the nitride coating layer similarly to its tribo-oxidation at the beginning of cutting (Figure 6a,f).

This process demonstrates the initial stage of the weakening of the upper nano-layers of the coating, while the wear velocity at the macro-level is still rather small (see Figure 1). The TEM examination of the very top nano-layer (about 20 nm thick, Figure 4) on the worn coating surface, shows the amorphous structure of the nitride coating layer [30,35]. This also indicates that all spontaneous processes associated with tribo-oxidation (such as phase de-composition and migration of desirable elements from the coating layer to the friction surface) are non-equilibrium as well.

This is yet another case of a nano-scale effect asserting the complexity of the self-organization process. 

## 5. Conclusions

This paper investigates complex tribological processes in PVD coatings, represented by an adaptive TiAlCrSiYN/TiAlCrN nano-multilayer PVD coating operating under extreme external stimuli (high temperature/heavy load wear conditions). This particular correlation leads to the ability of the adaptive coatings to sustain better under these extreme conditions. These complex processes are outlined in the following features:

(1) Accelerated adaptive response to extreme external stimuli at the very beginning of the running-in stage of wear. This is exhibited through the formation of a larger amount of protective surface tribo-films, which leads to a decrease in the wear values.
(2) Formation of synergistic complex tribo-oxides (such as mullites and garnets) at the beginning of wear process, which, in combination with sapphire, possess enhanced thermal barrier properties. This creates an effect where intensive heat flow that develops during cutting, minimally accumulates within the nano-layer of the tribo-films and is maximally reflected into the environment, providing enhanced thermal protection of the coating sub-layer.
(3) The evolution of protective oxide formation on the wear surface of multilayer nitride coatings was traced as being directly associated with their complex temporal behavior resulting from self-organization processes.

All of the processes outlined above occur at the nano-scale and interact in a highly sophisticated way, resulting in a significant reduction of entropy production during friction.

## References

[1] Fox-Rabinovich G., Totten G.E., Fox-Rabinovich G., Totten G.E. (2006). Self-Organization During Friction—Advanced Surface-Engineered Materials and Systems Design.

[2] Hanafi I., Khamlichi A., Cabrera F.M., Almansa E., Jabbouri A. (2012). Optimization of cutting conditions for sustainable machining of PEEK-CF30 using TiN tools. J. Clean. Prod..

[3] Fox-Rabinovich G.S., Gershman I.S., Yamamoto K., Aguirre M.H., Covelli D., Arif T., Aramesh M., Shalaby M.A., Veldhuis S. (2017). Surface/interface phenomena in nano-multilayer coating under severing tribological conditions. Surf. Interface Anal..

[4] Gupta S., Filimonov D., Palanisamy T., El-Raghy T., Barsoum M.W. (2007). Ta2AlC and Cr2AlC Ag-based composites—New solid lubricant materials for use over a wide temperature range against Ni-based superalloys and alumina. Wear.

[5] Voevodin A.A., Zabinski J.S. (2005). Nanocomposite and nanostructured tribological materials for space applications. Compos. Sci. Technol..

[6] Bouzakis K.-D., Michailidis N., Skordaris G., Bouzakis E., Biermann D., M’Saoubi R. (2012). Cutting with coated tools: Coating technologies, characterization methods and performance optimization. CIRP Ann..

[7] Wainstein D.L., Kovalev A.I. (2002). Fine determination of interatomic distances on surface using extended energy-loss fine structure (EELFS) data: Peculiarities of the technique. Surf. Interface Anal..

[8] Amiri M., Khonsari M.M. (2010). On the Thermodynamics of Friction and Wear―A Review. Entropy.

[9] Fox-Rabinovich G.S., Kovalev A.I., Aguirre M.H., Beake B.D., Yamamoto K., Veldhuis S.C., Endrino J.L., Wainstein D.L., Rashkovskiy A.Y. (2009). Design and performance of AlTiN and TiAlCrN PVD coatings for machining of hard to cut materials. Surf. Coat. Technol..

[10] Krzanowski J.E. (2004). Phase formation and phase separation in multiphase thin film hard coatings. Surf. Coat. Technol..

[11] Voevodin A.A., Fitz T.A., Hu J.J., Zabinski J.S. (2002). Nanocomposite tribological coatings with “chameleon” surface adaptation. J. Vac. Sci. Technol. A.

[12] Jacobson S., Hogmark S. (2009). Surface modifications in tribological contacts. Wear.

[13] Fox-Rabinovich G., Kovalev A., Veldhuis S., Yamamoto K., Endrino J.L., Gershman I.S., Rashkovskiy A., Aguirre M.H., Wainstein D.L. (2015). Spatio-temporal behaviour of atomic-scale tribo-ceramic films in adaptive surface engineered nano-materials. Sci. Rep..

[14] Aouadi S.M., Luster B., Kohli P., Muratore C., Voevodin A.A. (2009). Progress in the development of adaptive nitride-based coatings for high temperature tribological applications. Surf. Coat. Technol..

[15] Erdemir A. (2005). A crystal chemical approach to the formulation of self-lubricating nanocomposite coatings. Surf. Coat. Technol..

[16] Mandrino D., Podgornik B. (2017). XPS investigations of tribofilms formed on CrN coatings. Appl. Surf. Sci..

[17] Haque T., Morina A., Neville A., Kapadia R., Arrowsmith S. (2007). Non-ferrous coating/lubricant interactions in tribological contacts: Assessment of tribofilms. Tribol. Int..

[18] Fox-Rabinovich G.S., Yamamoto K., Beake B.D., Gershman I.S., Kovalev A.I., Veldhuis S.C., Aguirre M.H., Dosbaeva G., Endrino J.L. (2012). Hierarchical adaptive nanostructured PVD coatings for extreme tribological applications: The quest for nonequilibrium states and emergent behavior. Sci. Technol. Adv. Mater..

[19] Olofsson J., Gerth J., Nyberg H., Wiklund U., Jacobson S. (2011). On the influence from micro topography of PVD coatings on friction behaviour, material transfer and tribofilm formation. Wear.

[20] Suszko T., Gulbiński W., Morgiel J., Greczynski G., Dobruchowska E., Dłużewski P., Lu J., Hultman L. (2017). Amorphous FeCrNi/a-C:H coatings with self-organizednanotubular structure. Scr. Mater..

[21] Lehn J.-M. (2002). Toward Self-Organization and Complex Matter. Science.

[22] Keckes J., Daniel R., Mitterer C., Matko I., Sartory B., Koepf A., Weißenbacher R., Pitonak R. (2013). Self-organized periodic soft-hard nanolamellae in polycrystalline TiAlN thin films. Thin Solid Films.

[23] Yates F.E., Yates F.E., Garfinkel A., Walter D.O., Yates G.B. (1987). Self-Organizing Systems.

[24] Fox-Rabinovich G.S., Beake B.D., Yamamoto K., Aguirre M.H., Veldhuis S.C., Dosbaeva G., Elfizy A., Biksa A., Shuster L.S. (2010). Structure, properties and wear performance of nano-multilayered TiAlCrSiYN/TiAlCrN coatings during machining of Ni-based aerospace superalloys. Surf. Coat. Technol..

[25] Fox-Rabinovich G.S., Yamamoto K., Beake B.D., Kovalev A.I., Aguirre M.H., Veldhuis S.C., Dosbaeva G.K., Wainstein D.L., Biksa A., Rashkovskiy A. (2010). Emergent behavior of nano-multilayered coatings during dry high-speed machining of hardened tool steels. Surf. Coat. Technol..

[26] Kondepudi D., Prigogine L., Kondepudi D., Prigogine L. (2014). Modern Thermodynamics: From Heat Engines to Dissipative Structures.

[27] Shaw M. (2005). Metal Cutting Principles.

[28] Berent V.Y., Gershman I.S. (1990). Secondary structures on surfaces of sliding contacts at high current. 3. Mechanisms of formation, growth and destruction of secondary structures. J. Frict. Wear.

[29] Grasso S., Tsujii N., Jiang Q., Khaliq J., Maruyama S., Miranda M., Simpson K., Mori T., Reece M.J. (2013). Ultra low thermal conductivity of disordered layered p-type bismuth telluride. J. Mater. Chem. C.

[30] Fox-Rabinovich G., Kovalev A., Aguirre M.H., Yamamoto K., Veldhuis S., Gershman I., Rashkovskiy A., Endrino J.L., Beake B., Dosbaeva G. (2014). Evolution of self-organization in nano-structured PVD coatings under extreme tribological conditions. Appl. Surf. Sci..

[31] Kingery W.D. (1959). Thermal Conductivity: XIV, Conductivity of Multicomponent Systems. J. Am. Ceram. Soc..

[32] Cao X.Q., Vassen R., Stoever D. (2004). Ceramic materials for thermal barrier coatings. J. Eur. Ceram. Soc..

[33] Barsoum M., Barsoum M.W. (2002). Fundamentals of Ceramics.

[34] Yuan J., Yamamoto K., Covelli D., Tauhiduzzaman M., Arif T., Gershman I.S., Veldhuis S.C., Fox-Rabinovich G.S. (2016). Tribo-films control in adaptive TiAlCrSiYN/TiAlCrN multilayer PVD coating by accelerating the initial machining conditions. Surf. Coat. Technol..

[35] Watari K., Ishizaki K., Tsuchiya F. (1993). Phonon scattering and thermal conduction mechanisms of sintered aluminium nitride ceramics. J. Mater. Sci..

[36] Fox-Rabinovich G.S., Endrino J.L., Aguirre M.H., Beake B.D., Veldhuis S.C., Kovalev A.I., Gershman I.S., Yamamoto K., Losset Y., Wainstein D.L. (2012). Mechanism of adaptability for the nano-structured TiAlCrSiYN-based hard physical vapor deposition coatings under extreme frictional conditions. J. Appl. Phys..

[37] Wainstein D., Kovalev A. (2018). Tribooxidation as a Way to Improve the Wear Resistance of Cutting Tools. Coatings.

[38] Duminica F.-D., Belchi R., Libralesso L., Mercier D. (2018). Investigation of Cr(N)/DLC multilayer coatings elaborated by PVD for high wear resistance and low friction applications. Surf. Coat. Technol..

[39] Gustavsson F., Jacobson S. (2016). Diverse mechanisms of friction induced self-organisation into a low-friction material—An overview of WS2 tribofilm formation. Tribol. Int..

[40] AlAnazi F., Ghosh S., Dunnigan R., Gupta S. (2017). Synthesis and tribological behavior of novel Ag- and Bi-based composites reinforced with Ti_3_SiC_2_. Wear.

[41] Panjan P., Drnovšek A., Kovač J. (2018). Tribological aspects related to the morphology of PVD hard coatings. Surf. Coat. Technol..

